# Beyond Platinum Resistance: Reconsidering Platinum Sensitivity as a Dynamic Phenotype in Ovarian Cancer

**DOI:** 10.3390/cancers18203295

**Published:** 2026-10-13

**Authors:** Alicia A. Goyeneche, Adriana E. Rosato, Roberto R. Rosato, Carlos M. Telleria

**Affiliations:** 1 Cancer Research Program, Research Institute, McGill University Health Centre, Montreal, QC H4A 3J1, Canada; alicia.goyeneche@affiliate.mcgill.ca; 2 Center for Molecular Medicine, MaineHealth Institute for Research, Scarborough, Maine, ME 04074, USA; adriana.rosato@mainehealth.org (A.E.R.); roberto.rosato@mainehealth.org (R.R.R.); 3 Department of Pathology, Faculty of Medicine and Health Sciences, McGill University, Montreal, QC H3A 2B4, Canada; 4 Gerald Bronfman Department of Oncology, Faculty of Medicine and Health Sciences, McGill University, Montreal, QC H4A 3T2, Canada

**Keywords:** ovarian cancer, platinum (Pt) resistance, Pt sensitivity, Pt rechallenge, Pt resensitization, cisplatin, carboplatin, tumor evolution, biomarkers

## Abstract

Platinum-based chemotherapy remains a key treatment for epithelial ovarian cancer, but platinum resistance is a major cause of treatment failure and mortality. Traditionally, platinum resistance has been defined by the interval between the last platinum treatment and disease progression, with progression within six months generally classified as platinum-resistant. This six-month platinum-free interval has long provided a useful framework for treatment selection. However, growing preclinical and clinical evidence suggests that this interval should not be treated as a strict biological boundary. Platinum susceptibility can change as tumors evolve, with some tumors retaining residual sensitivity while others develop stable or potentially modifiable resistance mechanisms. These mechanisms include restoration of homologous recombination, altered DNA-damage tolerance, epigenetic regulation, changes in platinum transport and detoxification, adaptive survival signaling, and alterations in cell-death pathways. Some resistance mechanisms are relatively stable, whereas others may remain biologically plastic and potentially amenable to therapeutic modification. This distinction has important implications for platinum rechallenge. Responses to platinum reintroduction in selected patients previously classified as platinum-resistant indicate that the six-month interval does not fully capture the biology of platinum responsiveness. However, renewed clinical activity after rechallenge should not automatically be interpreted as biological resensitization. True resensitization requires evidence that a defined resistance mechanism has been modified and that this alteration increases platinum susceptibility. Preclinical studies have identified multiple strategies to modify platinum-resistance pathways, but clinical translation remains limited. This perspective therefore proposes that platinum resistance should be understood as a clinically defined phenotype encompassing biologically heterogeneous states, some of which may be stable and others potentially reversible. Recognizing this distinction has implications for biomarker development, treatment selection, trial design, and interpretation of platinum rechallenge. Prospective studies incorporating longitudinal tumor profiling and functional assessments of platinum susceptibility may ultimately identify patients in whom platinum activity can be therapeutically restored.

## Simple Summary

Ovarian cancer is a serious disease that often recurs after initial treatment. Platinum-based chemotherapy is a main treatment for ovarian cancer, and many patients initially respond well. However, the cancer may eventually become less responsive to these drugs, a condition commonly called platinum resistance. Ovarian cancer is often classified as platinum-resistant when the cancer returns within six months after the last platinum treatment. Although this six-month interval helps guide treatment decisions, it may not fully reflect how the cancer responds to treatment. Cancer cells can change over time, and their sensitivity to platinum drugs may increase or decrease during the course of the disease. Therefore, some patients considered to have platinum-resistant cancer may still respond to another platinum-based treatment. Recognizing that platinum sensitivity can change over time may improve how treatment response is assessed and help identify patients who could benefit from further platinum-based therapy.

## 1. Introduction

Ovarian cancer remains a significant global health burden, with substantial geographic variation in incidence and mortality. Between 1990 and 2021, the number of new cases worldwide rose from 159,096 to 298,876, while the global age-standardized incidence rate (ASIR; the number of new ovarian cancer cases expected per 100,000 women if all populations had the same standard age distribution) declined modestly from 7.22 to 6.71. During the same period, ovarian cancer-related deaths increased from 100,584 to 185,609, and global disability-adjusted life years (DALYs; a measure of the overall health burden caused by a disease that combines premature death and time lived with disability or reduced health, calculated as years of life lost plus years lived with disability) reached 5.16 million in 2021, indicating a substantial burden from both premature mortality and morbidity. South Asia recorded the highest number of incident cases and deaths, while China, India, and the United States accounted for approximately 35% of global new cases. Despite declining age-standardized incidence and mortality rates, the rising absolute burden highlights the continuing challenges in the prevention and treatment of ovarian cancer [1].

Platinum (Pt)-based chemotherapy remains a key systemic treatment for epithelial ovarian cancer. Cisplatin and carboplatin exert their cytotoxic effects primarily by forming DNA adducts and crosslinks, causing DNA damage that interferes with replication and transcription and can ultimately trigger cell death [2,3,4,5,6,7]. Despite major advances in surgery, molecular classification, maintenance therapies, antibody–drug conjugates, and targeted medications, Pt compounds remain important for managing recurrent disease.

Recurrent ovarian cancer has traditionally been categorized by the interval between completion of Pt therapy and subsequent disease progression. Recurrence more than six months after Pt treatment is generally classified as Pt-sensitive, whereas progression within six months is designated Pt-resistant. Progression during Pt treatment or shortly thereafter is often described as Pt-refractory [7,8,9,10]. This terminology remains clinically useful because, at the population level, the interval since the previous Pt treatment correlates with the probability of response to subsequent Pt therapy. However, the six-month threshold should not be interpreted as a precise biological boundary. Tumor response to Pt reflects interactions among tumor genetics, epigenetic regulation, DNA-repair capacity, drug transport, cellular stress responses, cell-death pathways, treatment history, the tumor microenvironment, and clonal evolution [8,9,10,11,12,13,14].

Tumors are also heterogeneous. Primary tumors may harbor multiple cellular populations with varying degrees of Pt susceptibility, and treatment can impose selective pressures that shift their relative abundance over time. Consequently, the phenotype observed at recurrence may reflect the tumor’s evolutionary state at that time rather than an irreversible property determined solely by the timing of progression.

This distinction has become increasingly relevant as Pt rechallenge is reconsidered in selected patients previously classified as Pt-resistant. Retrospective and real-world studies have reported clinically meaningful responses to Pt re-treatment in selected patients, including those with homologous recombination repair (HRR) deficiency—in this work, for simplicity, HRR status will be mostly used throughout to encompass homologous recombination deficiency (HRD), HRR proficiency/deficiency, and BRCA1/2 mutation or reversion status. These observations challenge the assumption that Pt-resistance necessarily represents a permanent loss of Pt susceptibility. However, they do not establish that the tumor has undergone biological resensitization.

Accordingly, three concepts should be distinguished. Clinical Pt resistance is a treatment-response classification based primarily on the timing of disease progression relative to prior Pt exposure. Biological Pt resistance refers to cellular or molecular mechanisms that reduce Pt effectiveness. Pt resensitization should be reserved for situations in which a resistance mechanism is demonstrably altered, and Pt susceptibility subsequently increases. The six-month Pt-free interval therefore remains a useful clinical descriptor, but it should not be regarded as an absolute biological definition of irreversible resistance.

Recent reviews have described mechanisms of Pt resistance and therapeutic strategies to improve treatment of Pt-resistant ovarian cancers (PROCs) [15,16,17], including the first phase II trial by Gilbert et al. [18] to evaluate the safety and preliminary efficacy of mirvetuximab soravtansine (MIRV), a first-in-class antibody–drug conjugate targeting the folate receptor α (FRα), combined with bevacizumab. However, this perspective differs from these reviews by integrating resistance and resensitization into a unified temporal framework in which Pt responsiveness is a dynamic phenotype that may evolve, diminish, and potentially recover during treatment.

## 2. The Six-Month Rule: Clinically Useful but Biologically Incomplete

The six-month Pt-free interval served as a practical clinical measure of treatment sensitivity. Earlier clinical studies showed that the likelihood of responding to subsequent Pt therapy decreases as the interval from the preceding Pt treatment shortens. Clinicians subsequently incorporated this classification into clinical-trial eligibility criteria, treatment algorithms, and clinical communication. Because the Pt-free interval is readily available and does not require molecular testing, it remains an attractive clinical variable. It has demonstrated prognostic and predictive associations with subsequent treatment outcomes [8,9,10].

Nevertheless, the six-month threshold is a classification convention rather than a precise biological marker. No specific molecular event occurs at six months that abruptly converts a tumor from Pt-sensitive to Pt-resistant. Resistance can emerge through multiple and overlapping processes, including enhanced DNA repair, restoration of homologous recombination, altered intracellular Pt exposure, detoxification, changes in cellular death thresholds, altered tolerance of DNA damage, epigenetic remodeling, and activation of prosurvival signaling pathways [2,4,19,20,21,22,23,24].

Genomic studies have further demonstrated substantial evolutionary changes in high-grade serous ovarian cancer (HGSOC), the predominant histologic subtype and a major contributor to ovarian cancer mortality [7]. Primary tumors may harbor multiple subclones, and treatment-induced selective pressure can alter tumor composition over time. Thus, the clinical phenotype observed at recurrence may reflect the tumor’s biological state at that point in its evolution rather than a permanently established characteristic.

The six-month rule should therefore not be considered incorrect. Rather, it should be recognized as incomplete. It provides a clinically useful estimate of the probability of benefit from subsequent Pt therapy. However, it does not, by itself, determine the molecular state of Pt susceptibility in an individual tumor.

## 3. Pt Resistance as an Evolving Phenotype

Pt resistance is mechanistically heterogeneous. The cytotoxic activity of Pt compounds depends on a series of interconnected processes, including drug delivery to the tumor, cellular uptake, intracellular trafficking, formation and persistence of DNA adducts, recognition and processing of DNA damage, cell-cycle responses, and execution of cell death [3,19,20]. Alterations at any of these levels can influence Pt susceptibility.

One important mechanism is the restoration of homologous recombination proficiency. In tumors with BRCA1 or BRCA2 deficiency, secondary mutations can restore the open reading frame and partially or completely restore homologous recombination function, thereby reducing sensitivity to Pt and Poly (ADP-ribose) polymerase (PARP) inhibitors [25,26]. Because these alterations are genetically encoded and can be transmitted to subsequent tumor-cell generations, such resistance may be relatively stable.

Other resistance mechanisms may be more plastic. Epigenetic regulation can alter the expression of DNA-repair genes, cell-death regulators, and drug-response pathways without necessarily changing the DNA sequence. Experimental studies show that pharmacological modulation of DNA methylation or histone acetylation can alter Pt sensitivity, providing a biological rationale for targeting specific resistant phenotypes [27,28,29,30,31].

Resistance can also involve adaptive survival signaling. Activation of PI3K/AKT/mTOR, JAK/STAT, stress-response pathways, and other prosurvival networks can enable tumor cells to tolerate DNA damage levels that would otherwise be lethal [32,33]. Similarly, changes in Pt transport and detoxification can reduce effective intracellular exposure [34]. These mechanisms are not mutually exclusive. A tumor may simultaneously exhibit enhanced DNA repair, increased tolerance of DNA damage, altered survival signaling, and changes in cell-death execution. Moreover, the relative contribution of individual mechanisms may shift during treatment.

**Figure 1:**
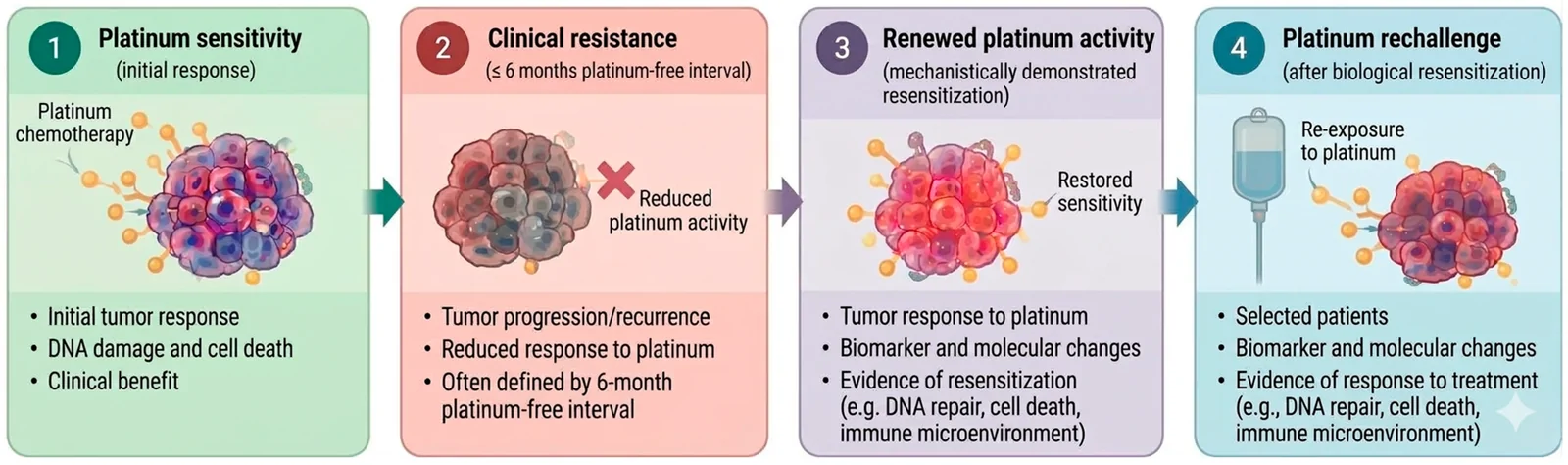
Clinical and biological framework for Pt resensitization. From Pt sensitivity (1) to clinical resistance (2), to biological resensitization to Pt (3), and ultimately to response to Pt rechallenge therapy (4). Arrows indicate the conceptual progression between stages, emphasizing the distinction between observed clinical response and experimentally supported biological resensitization. This conceptual figure was designed by the authors, with selected graphical elements generated using ChatGPT 6 (OpenAI).

The resulting heterogeneity is important. Tumors classified clinically as Pt-resistant may therefore represent distinct biological states, ranging from relatively stable resistance to partially plastic states in which residual Pt susceptibility persists. The clinically relevant question is consequently not whether Pt resistance can be universally reversed, but whether particular resistant tumors retain a biologically modifiable vulnerability that can be exploited therapeutically (Figure 1).

Within this dynamic framework, stable resistance denotes persistent loss of Pt responsiveness despite continued or repeated exposure, whereas residual susceptibility describes the persistence of a Pt-responsive subpopulation despite an overall resistant clinical phenotype. Plastic resistance refers to a reversible state in which tumor cells adapt to Pt exposure but retain the capacity to regain Pt sensitivity under altered selective conditions. Genuine resensitization should be distinguished from renewed clinical activity and ideally requires evidence that a previously resistant phenotype has been biologically reversed, ideally supported by experimental or molecular evidence where available. The literature supports measurable characteristics associated with these states, although no universally validated biomarker panel currently distinguishes all four in individual patients. Stable Pt resistance may be inferred from progression during or shortly after Pt therapy and from persistent biological alterations affecting DNA-damage repair, Pt transport, detoxification, or cell death [15]. Residual Pt susceptibility may be reflected by continued clinical benefit despite a short Pt-free interval, heterogeneous response, or response to subsequent Pt rechallenge; importantly, Pt response appears to vary continuously with the treatment-free interval rather than follow a strict 6-month biological threshold [35]. Plastic resistance is characterized experimentally by a potentially reversible drug-tolerant state involving adaptive changes in proliferation, stress responses, or cellular phenotype [36]. Finally, genuine Pt resensitization ideally requires restoration of Pt responsiveness together with evidence that an underlying resistance mechanism has been reversed or bypassed [37].

## 4. Pt Resensitization: A Distinct Biological Concept

A patient who initially progressed within six months of Pt treatment may later respond to another Pt-containing regimen. Such a response indicates that clinically meaningful Pt activity can occur after apparent resistance, but it does not explain why that activity has returned. Several explanations are possible. The six-month classification may have underestimated residual Pt susceptibility; intervening treatment may have reduced or eliminated resistant subclones; a previously minor Pt-sensitive population may have become dominant; the tumor microenvironment may have changed; or subsequent treatment may have altered selective pressures. Alternatively, an intervention may have directly modified a resistance mechanism, thereby increasing Pt susceptibility. The latter situation represents biological resensitization. A convincing demonstration of resensitization should ideally establish three elements: (1) a defined mechanism of Pt resistance; (2) an intervention that modifies or suppresses that mechanism; and (3) restoration or enhancement of Pt susceptibility attributable to the intervention. This distinction matters because a clinical response after Pt rechallenge does not necessarily reflect any of these mechanistic events. A response to Pt rechallenge indicates renewed clinical Pt activity; it does not, by itself, demonstrate biological resensitization. Thus, Pt rechallenge and Pt resensitization should not be treated as synonymous. Pt rechallenge is a clinical treatment strategy, whereas resensitization is a mechanistic biological hypothesis that requires evidence beyond tumor response alone.

## 5. Current Management of Pt-Resistant Ovarian Cancer (PROC)

The management of PROC has evolved substantially. Current approaches include non-Pt chemotherapy, antiangiogenic therapy, biomarker-directed antibody–drug conjugates, and glucocorticoid receptor-guided therapy (Table 1). Notably, PARP inhibitors are used primarily as maintenance after a complete or partial response to Pt chemotherapy, whereas patients who do not respond to Pt should not receive PARP inhibitor maintenance [38]. These treatments matter because therapeutic benefit in PROC does not necessarily require restoring Pt sensitivity. Many contemporary therapies exploit vulnerabilities independent of Pt response. Consequently, clinical benefit from a non-Pt treatment should not be interpreted as evidence that Pt resistance has been reversed. Conversely, the availability of effective non-Pt therapies provides an important comparator for studies evaluating Pt rechallenge or resensitization. A contemporary trial should not simply determine whether Pt can produce tumor shrinkage in patients previously classified as Pt-resistant. It should determine whether a Pt-based strategy provides clinically meaningful benefit compared with appropriate contemporary alternatives.

## 6. Preclinical Evidence for Restoration of Pt Susceptibility

A substantial body of experimental research supports the possibility that specific components of Pt resistance can be modified (Table 2). Epigenetic approaches provide some of the earliest evidence. DNA methylation inhibitors and histone deacetylase (HDAC) inhibitors can alter the expression of genes involved in DNA repair, cell-death pathways, and drug response. The DNA methyltransferase (DNMT) inhibitor Zebularine was reported to exert antimitogenic and chemosensitizing effects in ovarian cancer models [29]. Azacitidine, another DNMT inhibitor, increased Pt sensitivity in PROC cells [30], whereas valproic acid, an HDAC inhibitor, was reported to resensitize cisplatin-resistant ovarian cancer cells [31]. These observations indicate that at least some resistance phenotypes may be influenced by reversible transcriptional programs rather than by permanent genetic alterations alone.

Other studies have focused on downstream survival and DNA-damage-response pathways. For instance, HDAC4-regulated STAT1 signaling has been linked to Pt resistance [27]. Furthermore, MLN4924, an inhibitor of the NEDD8-activating enzyme, has been shown to enhance Pt sensitivity in preclinical models of ovarian cancer, primarily by inhibiting cullin neddylation and disrupting cullin-RING E3 ubiquitin ligase activity [52]. APR-246, which affects p53-associated pathways, has demonstrated activity against cisplatin-resistant ovarian cancer cells [53]. Inhibition of mTORC1/2 can also increase Pt sensitivity, in part by disrupting translation of transcripts involved in DNA-damage responses and cell survival [32,33]. Activation of FOXO3a has similarly been explored as a strategy to increase cellular sensitivity to cisplatin [54].

Other approaches have targeted Pt transport, DNA-damage tolerance, and prosurvival signaling. ATP7B, a membrane-associated P-type ATPase copper transporter involved in intracellular copper handling, has also been implicated in Pt resistance; researchers have investigated its silencing to restore Pt sensitivity in preclinical ovarian cancer models [34]. Chloroxine, a synthetic quinoline derivative with antimicrobial and antiseptic properties, has shown potential for repurposing to overcome Pt resistance [37]. Diacylglycerol kinase alpha (DGKA), which converts diacylglycerol (DAG) into phosphatidic acid (PA), promotes c-JUN–WEE1 signaling and has been implicated in Pt resistance; pharmacological inhibition of DGKA activity can enhance Pt sensitivity in preclinical ovarian cancer models [55]. Nanoparticle co-delivery of carboplatin and PF543, a potent, selective small-molecule inhibitor of sphingosine kinase 1, has also been reported to restore Pt sensitivity by inhibiting Pt-induced prosurvival signaling [56].

Regulation of cell death represents another potential point of intervention. We found that the gold complex auranofin, originally developed as an antirheumatic drug, sensitizes Pt-resistant epithelial ovarian cancer cells to cisplatin [57]. We also showed that the HIV protease inhibitor nelfinavir, when combined with cisplatin, produced synergistic activity against Pt-resistant ovarian cancer cells by inducing lytic cell death, increasing endoplasmic reticulum stress, enhancing DNA damage, reducing DNA repair, and impairing AKT survival signaling [58].

Taken together, these studies show that multiple components of Pt resistance can be manipulated experimentally. They provide biological plausibility for resistance-modifying strategies, although they do not establish that Pt resistance is universally reversible.

## 7. Clinical Evidence for Pt Rechallenge and Resensitization

Translating preclinical resensitization concepts into clinical practice has been markedly limited. Nevertheless, several clinical observations challenge the assumption that a patient who develops Pt-resistant disease can never benefit from Pt again. Early clinical experience already suggested that selected patients with apparently Pt-refractory disease could occasionally respond to carboplatin reintroduction [13]. These observations mattered because they showed that clinical Pt resistance was not synonymous with absolute Pt insensitivity.

Recent retrospective studies suggest that clinical Pt resistance does not invariably predict a complete absence of subsequent Pt activity. Valenza et al. reported an approximately 27% response rate in a selected group of 30 patients with PROC who had previously received non-Pt therapy; in this study, Pt resistance was defined as the interval from the last dose of the most recent line of Pt-based therapy to the date of disease progression or relapse after that line of therapy. Response was assessed using PFS, defined as the time from the first cycle of rechallenge with Pt salt monotherapy to disease progression or death from any cause, whichever occurred first; OS was defined as the time from the first cycle of rechallenge to death [50]. García-Illescas et al. similarly reported clinically meaningful activity in patients who had remained off Pt for at least 12 months and had received intervening non-Pt treatment, with responses observed in both BRCA-mutated and BRCA-wild-type tumors [12]. These findings prove that patients classified as Pt-resistant can, under selected circumstances, respond to subsequent Pt therapy. However, the selection characteristics of these cohorts are essential to interpretation. The García-Illescas study, for example, examined a population with a prolonged interval since prior Pt treatment, distinguishing these patients from those who progressed shortly after Pt exposure [12].

Yerram et al. [21] provided a larger real-world analysis that excluded patients with primary Pt-refractory disease and compared patients who received Pt rechallenge with those treated with non-Pt therapy. Among 2907 patients, 528 received Pt rechallenge, and 2379 received non-Pt treatment. After adjustment for measured covariates, Pt rechallenge was associated with longer real-world PFS and OS [21]. These data are clinically important but should not be interpreted as conclusive evidence of biological resensitization. Residual confounding is likely because treatment selection is not random. Patients selected for Pt rechallenge may have had more favorable disease characteristics, including slower progression, better performance status, fewer prior treatment lines, or longer intervals since previous Pt exposure.

The collective evidence therefore most strongly supports biological heterogeneity among patients classified as Pt-resistant. Some tumors may retain residual Pt susceptibility, whereas others may have acquired more stable resistance. Others may undergo changes in tumor composition that allow renewed Pt activity. BRCA status may contribute to this heterogeneity, but current evidence is insufficient to establish BRCA status as a standalone biomarker of Pt resensitization. The observation of Pt activity in both BRCA-mutated and BRCA-wild-type tumors in the García-Illescas cohort is therefore hypothesis-generating rather than definitive [12]. Most importantly, clinical activity following Pt rechallenge does not identify the mechanism responsible for that activity. Renewed response could represent genuine biological resensitization, expansion of a pre-existing Pt-sensitive clone, reduction in resistant populations, or changes in tumor composition. Thus, Pt rechallenge provides evidence of renewed clinical activity but not necessarily of mechanistically demonstrated resensitization.

## 8. Strategies That Prolong the Pt-Free Interval Are Not Necessarily Resensitization

Pt resensitization is sometimes conflated with simply extending the Pt-free interval. The underlying assumption is that withholding Pt for a sufficiently long interval might allow tumor cells to regain sensitivity. However, clinical evidence does not support time alone as a reliable resensitization strategy (Table 3).

The MITO-8 trial directly tested whether delaying Pt-based therapy in recurrent ovarian cancer would improve outcomes. Extending the Pt-free interval did not improve OS and led to worse PFS than immediate Pt treatment [59]. These conclusions argue against interpreting time off Pt as an intervention that reliably restores Pt sensitivity. Similarly, the INOVATYON/ENGOT-ov5 trial compared trabectedin/PLD followed by Pt with carboplatin/PLD in patients whose disease had progressed 6–12 months after prior Pt treatment [60]. The trial did not show a clinical advantage to delaying Pt with the alternative treatment sequence. These studies are important because they distinguish two concepts that are sometimes treated as equivalent. A longer Pt-free interval may correlate with a higher likelihood of subsequent Pt activity, but the interval itself does not demonstrate a biological reversal of resistance. Time is a clinical variable; resensitization is a biological process. Consequently, prolonging the Pt-free interval should not be considered evidence of resensitization unless an accompanying biological mechanism is demonstrated.

**Table 3:** Clinical studies evaluating Pt rechallenge, resensitization, or manipulation of the Pt-free interval.

Study	Design	Intervention	Principal Finding	Resensitization?
Kavanagh et al. [13]	Clinical study of Pt reintroduction in Pt-refractory epithelial ovarian cancer.	Carboplatin reintroduction.	Selected patients with clinically refractory disease could nevertheless respond to subsequent carboplatin.	No. Demonstrates residual clinical Pt activity but not mechanism reversal.
Fang et al. [61]	Phase I/pharmacodynamic study in recurrent PROC.	Decitabine + carboplatin.	Demonstrated feasibility and biological activity of combining hypomethylation with Pt.	Suggestive but not definitive. Provides pharmacodynamic rationale but does not establish mechanistically confirmed resensitization.
Fu et al. [62]	Phase Ib-IIa study in Pt-resistant/refractory ovarian cancer.	Azacitidine followed by carboplatin.	Explored reversal of Pt resistance through hypomethylation.	Suggestive but not definitive. Supports pharmacological modification of resistance.
Matei et al. [63]	Single-arm, nonrandomized phase II clinical trial.	Decitabine + carboplatin.	Provided clinical and molecular evidence (demethylation of MLH1, RASSF1A, HOXA10, and HOXA11) correlated positively with PFS, supporting epigenetic modification of Pt response.	Relatively strong translational support, but insufficient to establish a universal mechanism because there is no control arm.
Cottrill et al. [64]	Case report of recurrent Pt-refractory ovarian cancer.	RRx-001 followed by Pt doublet.	Reported a partial response following a proposed resensitization strategy.	No. Single-patient evidence is hypothesis-generating.
Glasspool et al. [65]	Randomized phase II trial in recurrences after 6–12 months of Pt (partially sensitive ovarian cancers).	Decitabine + carboplatin versus carboplatin. The trial was terminated early because of insufficient efficacy and difficulties with treatment delivery; adding decitabine appears to reduce rather than increase carboplatin efficacy.	Tested whether hypomethylation could enhance Pt activity.	No. Further evidence that interval manipulation alone is insufficient. Negative evidence on a strategy that hypothetically could have increased Pt sensitivity.
Holloway et al./VIRO-15 [66]	Phase II clinical study in PROC.	Olvimulogene nanivacirepvec (Olvi-Vec)-primed immunochemotherapy.	Evaluated a pharmacological approach intended to restore Pt responsiveness.	Suggestive but not definitive.
Marquina et al. [67]	Phase II trial testing the resensitization (clinical) with trabectedin in PROC.	Trabectedin followed by carboplatin or a carboplatin-based combination.	Demonstrated clinical activity in a Pt-resistant population.	No direct proof of resensitization. Did not identify a biological reversal of Pt resistance.
Yu et al. [68]	Translational/clinical analysis of Olvi-Vec.	Olvi-Vec followed by Pt-based treatment.	Proposed increased Pt sensitivity followed by oncolytic immunotherapy.	Suggestive. Requires mechanistically controlled prospective validation.
Holloway et al. [69]	Phase III clinical trial framework.	Olvi-Vec followed by Pt-doublet chemotherapy and bevacizumab versus standard therapy.	Prospectively tested a resistance-modifying treatment sequence.	Not automatically. Clinical benefit must be distinguished from mechanistic proof of Pt resensitization.
Pignata et al./MITO-8 [59]	Randomized phase III trial.	Delayed versus immediate Pt-based therapy.	Prolonging the Pt-free interval did not improve OS and resulted in inferior PFS.	No. Provides important evidence that time away from Pt alone is not resensitization.
Colombo et al./INOVATYON [60]	Randomized phase III trial in recurrence 6–12 months after Pt.	Trabectedin/PLD followed by Pt versus carboplatin/PLD.	Did not establish improved outcomes from delaying Pt through an alternative sequence.	No. Further evidence that interval manipulation alone is insufficient. Negative evidence on a strategy that hypothetically could have increased Pt sensitivity.
Valenza et al. [50]	Retrospective cohort of Pt-resistant ovarian carcinoma.	Pt rechallenge.	Demonstrated clinically meaningful activity in selected PROC patients.	No. Clinical rechallenge activity without proof of mechanism reversal.
Yerram et al. [21]	Large real-world cohort of PROC.	Pt rechallenge versus non-Pt treatment.	Pt rechallenge was associated with improved adjusted real-world PFS and OS.	No. Retrospective association requires prospective validation and molecular characterization.

Abbreviations: PROC, Pt-Resistant Ovarian Cancer; PFS, Progression-Free Survival; OS, Overall Survival; PLD, Pegylated Liposomal Doxorubicin; Olvi-Vec, Olvimulogene Nanivacirepvec; MLH1, MutL homolog 1; RASSF1A, Ras Association Family 1 Isoform A; HOXA10, Homeobox A10; HOXA11, Homeobox A11.

## 9. A Dynamic Model of Pt Sensitivity

**Figure 2:**
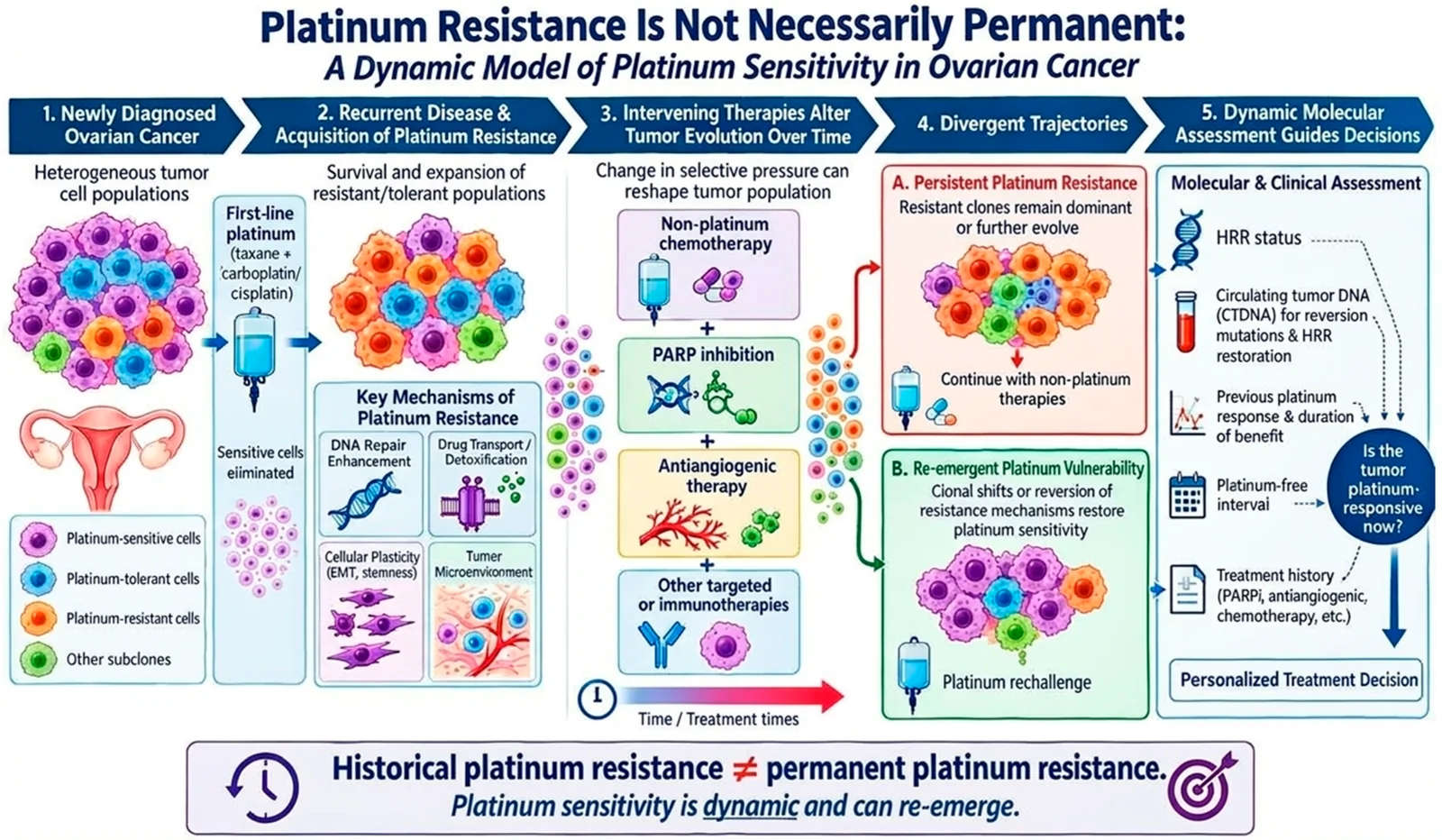
Proposed model illustrating the changing nature of Pt sensitivity in ovarian cancer. (1) Newly diagnosed ovarian cancer: primary tumors comprise heterogeneous cellular populations with variable Pt sensitivity, including Pt-sensitive, Pt-tolerant, and intrinsically resistant subpopulations. (2) Recurrent disease and acquisition of Pt resistance: following first-line Pt-based chemotherapy, selective pressure eliminates sensitive cells, while resistant or tolerant populations may persist and expand through means involving altered DNA damage responses and repair, drug transport and detoxification, cellular plasticity and epithelial–mesenchymal transition, and interactions with the tumor microenvironment. (3) Intervening therapies alter tumor evolution: subsequent non-Pt treatments—including cytotoxic chemotherapy, PARP inhibition, antiangiogenic therapy, and other targeted or immune-based approaches—may further modify the composition and biological state of the tumor over time. (4) Divergent trajectories: such evolutionary changes may result in either persistent Pt resistance, characterized by continued dependence on non-Pt therapies, or in a state of re-emergent Pt vulnerability, in which previously resistant tumors regain Pt sensitivity. (5) Dynamic molecular assessment: the potential for Pt rechallenge should therefore be considered prospectively using multiple clinical and biological factors, including HRR status, circulating tumor DNA and other biomarkers of molecular evolution, previous Pt response and its duration, Pt-free interval, and intervening treatment history. This system suggests that historical classification as Pt-resistant should not be treated as a permanent biological state and that Pt sensitivity may evolve over time and potentially re-emerge, supporting individualized consideration of Pt rechallenge. Abbreviations: EMT, Epithelial–Mesenchymal Transition; HRR, Homologous Recombination Repair; PARPi, PARP inhibitor; CTDNA, Circulating Tumor DNA. Conceptual figure designed by the authors, with selected graphical elements generated using ChatGPT 6 (OpenAI).

The evidence summarized above supports a model in which tumors clinically classified as Pt-resistant can occupy distinct biological states with varying degrees of residual Pt susceptibility (Figure 2). However, the links between the proposed assessments and treatment decisions require further validation.

At one end are tumors with relatively stable, mechanistically entrenched resistance. Examples include tumors in which genetic alterations restore homologous recombination or otherwise permanently modify determinants of Pt sensitivity. In such tumors, simply waiting longer or reintroducing Pt may have limited biological effect. At the other end are tumors in which resistance is driven predominantly by adaptive or plastic mechanisms. These may include reversible transcriptional programs, altered survival signaling, DNA-damage tolerance, metabolic adaptation, or regulated cell-death pathways. In these settings, the resistant phenotype may be more amenable to therapeutic modification. Between these extremes lies an important intermediate group. These tumors may exhibit heterogeneous clonal composition, partial resistance, or transient adaptations. They may retain sufficient Pt susceptibility to respond to subsequent Pt therapy.

The six-month definition captures only one component of this model. A patient progressing five months after Pt and another progressing seven months after Pt may share more biological characteristics than the clinical classification suggests. Conversely, two patients with identical progression intervals may have entirely different resistance mechanisms. The more informative biological question is therefore not simply, “Is the tumor Pt-resistant?” but rather, “What mechanism currently limits Pt effectiveness, and is that mechanism stable or potentially modifiable?” This conceptual shift has immediate implications for biomarker development, patient selection, and clinical trial design.

## 10. Biomarkers for a Dynamic Model of Pt Sensitivity

If Pt susceptibility can change during tumor evolution, biomarkers should ideally capture the tumor’s current biological state rather than relying exclusively on historical characteristics. HRR status remains an important determinant of Pt response, but static genomic classifications have limitations when tumors evolve under treatment. Functional assays may provide complementary information by assessing tumor cells’ current capacity for homologous recombination rather than relying solely on historical genomic alterations. Researchers have investigated functional homologous recombination assays as predictors of chemotherapy response and clinical outcome in ovarian cancer [70]. Functional RAD51 assays—RAD51 recombinase is a central protein in homologous recombination-mediated DNA repair, particularly the repair of DNA double-strand breaks [71]—are similarly being evaluated as tools to identify HRR status [72]. These approaches may be particularly relevant to a dynamic model because they can provide information about the tumor’s functional state at the time of treatment. Longitudinal assessment may be even more informative. Serial tumor sampling and circulating tumor DNA could identify the emergence of BRCA reversion mutations, changes in homologous recombination function, and alterations in other resistance-associated pathways. A tumor undergoing dynamic or incomplete restoration of HRR may represent a different therapeutic opportunity from one that has stably acquired proficient DNA-repair capacity. The same principle could apply to non-genetic mechanisms. Dynamic assessment of DNA-damage responses, cell-death signaling, AKT/mTOR activity, epigenetic states, drug transport, or other resistance-associated pathways could potentially identify tumors in which resistance remains pharmacologically modifiable. The biomarker strategy should therefore move progressively from static prediction toward longitudinal characterization of treatment state.

## 11. Consequences for Clinical-Trial Design

Several principles follow from this system. First, do not define Pt resensitization solely by tumor response after Pt rechallenge. Such responses are clinically meaningful but mechanistically nonspecific. If resensitization is the therapeutic objective, trials should incorporate biomarkers or functional endpoints that demonstrate modification of the resistance mechanism. Second, evaluate resistance-modifying interventions longitudinally. Whenever feasible, studies should characterize tumors before and after the intervention using paired tumor samples, circulating tumor DNA, functional assays, or other molecular measures. The objective should be to determine whether the resistance state has changed. Third, stratify patients by the biological context in which resistance developed. Primary Pt-refractory disease, early and later acquired Pt resistance, BRCA-reverted tumors with restored homologous recombination, HRR-proficient tumors, and tumors in which adaptive survival signaling predominates should not necessarily be considered biologically equivalent. Fourth, trial design should incorporate contemporary non-Pt therapies. The clinically relevant question is whether a resistance-modifying Pt strategy improves meaningful outcomes compared with effective alternatives, rather than simply whether Pt can produce responses in a historically Pt-resistant population. Fifth, eligibility criteria should not rely exclusively on the six-month cutoff. Patients progressing shortly before six months might be appropriate candidates for a resensitization trial if a biomarker identifies a potentially reversible resistance mechanism. In contrast, patients progressing after six months should not automatically be assumed to have biologically Pt-sensitive disease; identifying biomarkers of patient resensitization to Pt is essential for patient selection. Finally, trials should distinguish between renewed Pt activity and biological resensitization in their predefined endpoints. This distinction would improve interpretation throughout studies and prevent clinicians from using response as a surrogate for mechanistic reversal without supporting evidence.

## 12. Limitations of the Current Evidence

Several limitations prevent definitive conclusions about Pt resistance reversibility. First, much of the evidence supporting Pt resensitization remains preclinical. Experimental models can show that a resistance-associated pathway is pharmacologically modifiable, but successful preclinical reversal in vitro or in vivo does not guarantee clinically meaningful benefit. Second, much of the clinical evidence for Pt rechallenge is retrospective and therefore vulnerable to selection bias and residual confounding. Patients selected for rechallenge may have better performance status, slower disease kinetics, fewer prior lines of treatment, longer treatment-free intervals, or other favorable characteristics. Third, the definition of resensitization remains inconsistent. Some studies use a subsequent response to Pt after an intervening treatment as evidence of resensitization, whereas others require pharmacodynamic, molecular, or mechanistic evidence. This variation complicates comparisons across studies. Fourth, ovarian cancer is biologically heterogeneous. Resistance mechanisms may differ substantially between patients and may coexist within the same tumor. A single resistance-modifying treatment is therefore unlikely to reverse every form of Pt resistance. Fifth, longitudinal sampling remains uncommon. Without measurements before and after an intervention, it is difficult to determine whether a subsequent Pt response indicates genuine reversal of resistance, selection of pre-existing Pt-sensitive cells, residual susceptibility, or another change in tumor biology.

Consequently, the conventional 6-month Pt-free interval remains a clinically useful definition of Pt resistance and should not be abandoned at this time. Although emerging evidence suggests that Pt responsiveness may be dynamic and that resensitization can occur, much of the mechanistic evidence for true Pt resensitization remains preclinical. Thus, the 6-month criterion remains appropriate for clinical classification, while its limitations as a biological measure of irreversible resistance warrant further investigation.

However, the therapeutic landscape for PROC is evolving rapidly. Contemporary treatments, including biomarker-directed antibody–drug conjugates and other targeted approaches, such as combining a GR antagonist with nab-paclitaxel, offer increasingly relevant alternatives to conventional chemotherapy. Any future Pt-resensitization strategy should therefore demonstrate meaningful clinical benefit relative to contemporary standards.

## 13. Unresolved Gaps and Future Directions

Despite extensive literature on the molecular mechanisms and therapeutic strategies underlying Pt resistance, several key questions remain unresolved. In particular, current clinical frameworks do not fully account for the possibility that Pt responsiveness may be heterogeneous, dynamic, and reversible. The Pt-free interval (PFI) remains an important clinical parameter, but it is not a biological boundary. No validated framework or molecular or functional biomarker currently distinguishes stable Pt resistance from residual Pt susceptibility, plastic resistance, or genuine biological resensitization. Consequently, selecting patients with PROC for Pt rechallenge remains largely clinical, incorporating factors such as prior response to Pt, duration of the PFI, pattern and tempo of recurrence, prior treatment exposure, and overall clinical context. Although BRCA1/2 alterations, HRR deficiency, and other molecular features are biologically plausible candidates for predictive biomarker development, none has been clinically validated to prospectively identify patients with Pt-resistant disease who are likely to benefit from Pt rechallenge. Future studies should therefore move beyond the PFI as a binary measure and integrate longitudinal clinical response with molecular and functional assessments of tumor sensitivity. Such studies should address two distinct objectives: identifying patients with residual Pt susceptibility who may benefit from rechallenge, and determining whether an intervention has genuinely modified a resistance mechanism and restored Pt responsiveness.

The six-month Pt-free interval remains a useful clinical classification for recurrent ovarian cancer, but it should not be treated as a fixed biological boundary. The probability of subsequent Pt activity changes over time, yet the underlying biological determinants of that activity are heterogeneous and may evolve during treatment. Patients classified as Pt-resistant can occasionally respond to Pt rechallenge, indicating that the clinical classification does not capture the full range of Pt susceptibility. However, renewed clinical activity should not automatically be interpreted as biological resensitization. A subsequent Pt response may reflect residual susceptibility, clonal selection, changes in tumor composition, or genuine modification of a resistance mechanism. Preclinical studies provide strong evidence that multiple components of Pt resistance—including epigenetic regulation, DNA-damage responses, damage tolerance, survival signaling, Pt transport, and cell-death execution—can be experimentally modified. These findings establish biological plausibility for resistance-modifying strategies but do not demonstrate that Pt resistance is universally reversible. A convincing demonstration of Pt resensitization should therefore establish a defined resistance mechanism, show that an intervention modifies it, and confirm that the modification increases Pt susceptibility. Longitudinal functional assays, tumor sequencing, circulating tumor DNA, and paired molecular profiling may make such studies increasingly feasible.

## 14. Conclusions

The six-month PFI remains a useful clinical benchmark for classifying Pt response and guiding treatment decisions in patients with ovarian cancer. However, it does not strictly distinguish Pt-sensitive from Pt-resistant disease; rather, it marks the time from Pt treatment to disease progression. Clinicians use this threshold to stratify patients and select subsequent therapies, but it cannot fully capture the molecular basis of resistance, tumor heterogeneity, or treatment-induced changes in Pt sensitivity. Resistance can range from stable to more adaptable states, in which sensitivity might be temporarily restored. Therefore, the six-month cutoff should be viewed as an informative clinical marker rather than a definitive biological boundary. The therapeutic question should therefore shift from simply asking when Pt can be given again to determining whether, why, and in whom Pt activity can be restored.

## Figures and Tables

**Table 1:** Contemporary management of PROC and implications for Pt sensitivity.

Therapeutic Approach	Current Clinical Role	Relationship to Pt Sensitivity	Conceptual Implication	References
PLD, paclitaxel, topotecan, and gemcitabine in appropriate patients	Single-agent non-Pt chemotherapy. PLD; inhibits topoisomerase II, causing DNA damage. Paclitaxel; disrupts microtubules and prevents mitotic division. Topotecan; inhibits topoisomerase I, causing DNA single-strand breaks. Gemcitabine; pyrimidine nucleoside analog that inhibits DNA synthesis.	These approaches do not require restoration of Pt sensitivity and are generally used when Pt rechallenge is considered inappropriate or unlikely to provide sufficient benefit.	Their use reflects the clinical classification of disease as Pt-resistant but does not establish permanent loss of Pt susceptibility. Avoiding Pt is a therapeutic decision based on the probability of benefit, not proof of irreversible biological resistance.	[3,5,39,40,41,42,43]
Bevacizumab + chemotherapy	Established option for selected patients with Pt-resistant recurrent ovarian cancer; it can improve response and PFS compared with chemotherapy alone. Bevacizumab inhibits tumor angiogenesis and improves the delivery and distribution of chemotherapy within the tumor.	Activity is achieved without reintroducing Pt.	Benefit from a non-Pt treatment should not be interpreted as evidence that Pt sensitivity has been eliminated. Conversely, subsequent Pt activity after bevacizumab-containing therapy should not automatically be interpreted as resensitization.	[44,45]
Mirvetuximab soravtansine	Biomarker-directed treatment for patients with FRα-positive, PROC. Receptor-mediated internalization releases the microtubule inhibitor DM4 intracellularly, resulting in mitotic arrest and cell death, with additional bystander killing through membrane-permeable DM4 metabolites.	Therapeutic activity is based on FRα expression and antibody–drug conjugate delivery rather than restoration of Pt susceptibility.	Demonstrates that PROC is biologically heterogeneous and can contain actionable vulnerabilities independent of Pt response.	[18,46,47]
Relacorilant + nab-paclitaxel	Treatment strategy using a GR antagonist blocking the cell survival effect of glucocorticoids; for selected patients with PROC.	Does not require restoration of Pt sensitivity.	Provides a non-Pt comparator for future Pt-rechallenge or resensitization trials. Any proposed Pt-resensitizing strategy should demonstrate clinically meaningful benefit relative to effective alternatives.	[48,49]
Pt rechallenge as a selective clinical strategy	Pt may be reconsidered in selected patients despite a previous clinical classification of Pt-resistant disease, particularly when disease biology and treatment history suggest potential residual Pt activity. May select Pt-sensitive clones, attack cells with restored HRR, or attack cells with Pt plasticity.	A response after Pt reintroduction demonstrates clinically relevant Pt susceptibility at the time of rechallenge but does not establish reversal of the original resistance mechanism.	Pt rechallenge should be regarded as a clinical treatment strategy, not synonymous with biological resensitization.	[12,13,21,50]
Alternative non-Pt treatment before subsequent Pt	Sequential non-Pt treatment may control disease before considering subsequent Pt-based therapy in selected patients. Pt may attack cells that were sensitive and repopulate, or cells that were tolerant and re-enter the cell cycle.	Subsequent Pt activity may reflect residual sensitivity, changes in tumor composition, treatment selection, or biologically mediated resistance.	The observation that Pt can remain active after intervening non-Pt therapy supports dynamic Pt responsiveness but does not establish that the intervening treatment caused molecular resensitization.	[12,13,21,50]
Pt-free interval prolongation as an isolated strategy	Delaying Pt exposure to increase the interval between Pt treatments, potentially restoring subsequent Pt activity.	Randomized evidence does not demonstrate that increasing the interval alone reliably restores Pt sensitivity.	This represents an important negative-control concept: time away from Pt should not be equated with biological reversal of resistance.	[10]
Paclitaxel + pembrolizumab, with or without bevacizumab. The trial enrolled patients who were clinically Pt-resistant at trial entry, with 1–2 prior systemic lines, ≥1 Pt-based regimen, and Pt-resistant progression within 6 months of the last Pt treatment. Patients were enrolled regardless of PD-L1 expression, including those with PD-L1 CPS > 1, <10, ≥10, and ≥1. The regulatory indication is defined as treatment of patients with PD-L1 CPS ≥1 rather than the entire trial population.	Increases in PFS and OS in clinically defined Pt-resistant recurrent disease.	Does not demonstrate Pt resensitization. Does not provide a biomarker for selecting patients for Pt rechallenge.	Benefit from a non-Pt treatment should not be interpreted as evidence that Pt sensitivity has been eliminated.	[51]

Abbreviations: FRα, Folate Receptor Alpha; PLD, Pegylated Liposomal Doxorubicin; PFS, Progression-Free Survival; OS, Overall Survival; PROC, Pt-Resistant Ovarian Cancer; GR, Glucocorticoid Receptor; Pembrolizumab, PD-1 inhibitor; CPS, Combined Positive Score (a measure of PD-L1 expression in tumor tissue calculated as: (number of PD-L1–positive tumor cells + PD-L1–positive immune cells) ÷ (total number of viable tumor cells) × 100. Nab-Paclitaxel; Nanoparticle Albumin-Bound Paclitaxel.

**Table 2:** Representative preclinical evidence supporting potential Pt resensitization.

Mechanistic Strategy	Resistance Mechanism/Pathway Addressed	Experimental Finding	Interpretation
Zebularine [29]	DNA methylation and transcriptional regulation.	Increased chemosensitivity in ovarian cancer models.	Supports the possibility that some resistance phenotypes are epigenetically plastic.
Azacitidine [30]	Aberrant DNA methylation.	Increased sensitivity of PROC cells to carboplatin.	Provides a mechanistic rationale for epigenetic modification of resistance.
Valproic acid [31]	Histone deacetylase activity.	Resensitized cisplatin-resistant ovarian cancer cells.	Supports reversible transcriptional regulation as one component of resistance.
HDAC4/STAT1 pathway [27]	Adaptive transcriptional signaling.	HDAC4-regulated STAT1 activation was associated with Pt resistance.	Identifies a potentially modifiable signaling mechanism.
MLN4924 [52]	Neddylation, proteostasis, and DNA-damage responses.	Increased Pt sensitivity in preclinical ovarian cancer models.	Indicates that cellular stress and DNA-damage responses can be therapeutically altered.
APR-246 [53]	p53-associated cell-death signaling.	Increased cisplatin sensitivity in ovarian cancer models.	Supports restoration of cell-death competence as a potential resistance-modifying strategy.
mTORC1/2 inhibition [32,33]	Survival signaling and selective translation.	Increased sensitivity in Pt-resistant ovarian cancer models.	Demonstrates that adaptive survival programs can contribute to resistance.
FOXO3a activation [54]	Transcriptional control of survival.	Increased cisplatin susceptibility in ovarian cancer models.	Supports modulation of stress-response transcriptional states.
ATP7B targeting [34]	Pt transport and detoxification.	Targeting altered Pt handling affected Pt response in ovarian carcinoma models.	Indicates that intracellular Pt handling may be therapeutically modifiable.
Chloroxine [37]	DNA-damage tolerance.	Restored Pt sensitivity in HGSOC models.	Provides evidence that damage tolerance can be therapeutically targeted.
DGKA pathway inhibition [55]	c-JUN-WEE1 signaling and cell-cycle control.	DGKA was implicated in Pt resistance.	Identifies a potentially actionable resistance-associated signaling circuit.
PF543 + carboplatin [56]	Pt-induced prosurvival signaling.	Restored Pt sensitivity in ovarian cancer models.	Supports targeting adaptive survival responses accompanying Pt exposure.
Auranofin + cisplatin [57]	Redox and prosurvival signaling.	Auranofin sensitized cisplatin-resistant ovarian cancer cells.	Supports pharmacological modification of resistant cellular states.
Nelfinavir + cisplatin [58]	AKT signaling, DNA damage/repair, and cell-death execution.	Synergistic killing involving caspase-8/caspase-3/GSDME-associated lytic death.	Provides an example in which pharmacological pathway modulation increases cisplatin susceptibility in resistant cells.

Abbreviations: HDAC4, Histone Deacetylase 4; STAT1, Signal Transducer and Activator of Transcription 1; mTOR, Mechanistic Target of Rapamycin; FOXO3, Forkhead Box O3; ATP7B, ATPase Copper Transporting Beta; DGKA, Diacylglycerol Kinase Alpha; GSDME, Gasdermin E; HGSOC, High-Grade Serous Ovarian Cancer.

## Data Availability

No new data were created or analyzed in this study. Data sharing is not applicable to this article.
